# Impact of Load Variation on Lower Limb Joint Torque during Overhead Squats

**DOI:** 10.5114/jhk/195877

**Published:** 2025-10-01

**Authors:** Yusuke Ikeda, Masaki Kawabe, Tetsuya Hisamitsu

**Affiliations:** 1 Department of Physical Education, Nippon Sports Science University, Yokohama, Japan.; 2 Training Coach, Yokohama Shogyo High School, Yokohama, Japan.; 3 Itoman Swimming Nishinomiya, Nishinomiya, Japan.

**Keywords:** kinetics, kinematics, training, strength, flexibility

## Abstract

The purpose of this study was to examine the kinematic and kinetic changes in overhead squats (OHSs) with increasing loads and clarify the relationship between OHS movement and shoulder joint flexibility. Fourteen male college students randomly performed OHSs using 20% of their body mass (BM), 40% of their BM, and a plastic pipe (no load). The motion and ground reaction forces during OHSs were recorded. The relative peak torque of the hip under the 20% and 40% BM conditions during both negative and positive phases of the OHS was significantly greater than that under the no-load condition, while the relative peak torque of the knee did not increase. The relative peak torque of the hip was negatively correlated with the angles of the lower trunk at the lowest point of the center of mass (CM). In relation to the movement of the OHS and shoulder joint flexibility, a correlation was observed between the angle of the lower trunk at the lowest point of the CM and the shoulder flexibility test score (r = 0.561–0.598, p < 0.05), suggesting that a flexible shoulder joint could lead to the lower trunk leaning forward during the OHS. These results reveal that in the overhead squat, where the relative load to body weight is smaller compared to the normal squat, an increase in the load leads to an increase in hip torque without a corresponding increase in knee torque. Furthermore, the increase in hip torque is influenced by the angle of the lower trunk.

## Introduction

The squat exercise is one of the most fundamental training methods for enhancing lower extremity and trunk strength. Several studies have suggested that the squatting posture and neuromuscular coordination are similar to those required in many common activities, enabling a greater transfer of lower extremity strength acquired through squatting to these activities (Layer et al., 2018; Wilson et al., 1996; Wirth et al., 2016). Furthermore, several studies have documented improvements in jump performance (Arabatzi et al., 2010; Augustsson et al., 1998; Chelly et al., 2009; Christou et al., 2006) and sprinting ability (Seitz et al., 2014; Styles et al., 2016) resulting from strength training interventions incorporating squat exercises.

Several significant findings have been revealed about joint kinetics during squats. One study found that restricting the squat to prevent the knees from moving anteriorly past the toes significantly increased the hip torque compared to a normal squat (Fry et al., 2003). Another study indicated that increasing the stance width during squats generated greater knee and hip torque (Escamilla, 2001). Based on these suggestions, strength and conditioning coaches, trainers, and therapists must understand the alterations in muscle activity and kinematic and kinetic outcomes during squat exercises, particularly concerning changes in the posture (Aspe and Swinton, 2014; Gullett et al., 2009), depth of movement (Caterisano et al., 2002; Kasahara et al., 2024; McKean et al., 2010), load variation (Suchomel et al., 2024), and stance width (Escamilla et al., 2001). This understanding is essential for effective implementation of training and rehabilitation programs.

The overhead squat (OHS) is a variation of the squat exercise commonly used for strength training and injury screening. It was developed as one of the tools for assessing global movement patterns (Butler et al., 2010; Kiesel et al., 2011), referred to as the Functional Movement ScreenTM (FMSTM). The FMSTM comprises seven tests: the overhead squat, the hurdle step, the inline lunge, shoulder mobility, the active straight-leg raise, the trunk stability push-up, and rotary stability. Each test is scored on a scale from 0 to 3 (Cook et al., 2014). Individual scores are typically combined into composite scores, and a cutoff score of less than 14 has been commonly used to assess injury risk in athletes and tactical populations (Garrison et al., 2015; Heredia et al., 2021). Butler et al. (2010) compared joint angles and joint moments among three groups (Groups 1 to 3) with different FMS scores on the OHS. They observed that Group 3 in the OHS exhibited greater dorsiflexion excursion, a higher peak knee extension moment, and a greater peak hip extension moment than Group 1 did. This suggested that joint kinematic and kinetic factors influence movement and assessment during OHSs. However, it remains unclear whether changes in the load during the OHS or flexibility of participants’ shoulder joints and lower extremity affect OHS movement.

Only few studies have focused on the effects of OHS training, although it is commonly introduced in physical training programs for different types of athletes. Regarding golfers’ skills and physical training, Langdown et al. (2023) reported no significant changes in 3D golf swing kinematics despite a significant improvement in the OHS thigh angle within the intervention group after an 8-week training intervention. They concluded that using the OHS to understand the cause of postural loss during a golf swing was not recommended. However, Rose (2020) suggested that OHS performance was a valuable predictor of posture loss in golf swings. Regarding the relationship between sports performance and movement in the OHS, the kinematic and kinetic factors of the OHS are poorly understood. To design an effective strength training program to enhance athletic performance, strength, and conditioning, coaches, athletic trainers, and therapists must understand the changes in muscle activity and the relationships between flexibility and movement during the OHS. Thus, the features of OHS movement should be elucidated before considering its relationship with sports performance.

This study aimed to examine changes in the hip, knee, and ankle joint torque as the load increased while maintaining the same motion speed, depth, and stance width during the OHS. Additionally, we investigated the relationship between the kinematic and kinetic variables of the OHS and shoulder joint and lower extremity flexibility. We hypothesized that the torque at the hip, knee, and ankle joints would increase as the load during the OHS increased. We also hypothesized that shoulder flexibility, which was measured by a flexibility test, would affect the joint torque in the lower extremities during the OHS.

## Methods

### Participants

Fourteen male college students (age: 20.14 ± 0.8 years, body height: 1.72 ± 0.06 m, body mass: 70.1 ± 7.03 kg) who belonged to the faculty of the Department of Health and Sports participated in this study. All the participants regularly engaged in sports activities and were physically healthy. The Ethics Committee of the Niigata University of Health and Welfare approved this study (protocol code: 950-3198; approval date: 17 January 2023). Participants were fully informed of the experimental purpose and procedures of the present study, after which they provided signed informed consent.

### Measures

The motion speed of the OHSs was controlled using a tempo device (FINIS, Inc.). The duration of the motion during both the descending and ascending phases of the OHS was approximately 4 s. The motion of the OHSs was recorded in the sagittal plane for motion analysis using a digital video camera (GC-LJ20B, SPORTS SENSING Co., Fukuoka, Japan) placed perpendicularly to the participants' motion plane. The distance from the digital video camera to the participant on the force plate was approximately 12 m. A digitizing system (FrameDIAS V, DKH, Inc., Itabashi-ku, Tokyo, Japan) was used to manually digitize 14 points on the body (the head’s vertex, ear hole, superior margin of the sternum, acromion, lateral humeral epicondyle, styloid process of the wrist, metacarpophalangeal joint, inferior end of the rib, GT, lateral epicondyle of the femur, lateral malleolus, calcanei, hallux, and toe tip). The digitization rate was set to 60 Hz. In this study, we assumed that the movement of both the arms and legs of participants during the OHS was symmetrical; therefore, the left side of participants’ limbs recorded by the digital video camera was digitized. Two horizontal control points were used to obtain the two-dimensional coordinates. The horizontal-to-vertical ratio of the video image was evaluated in advance to calculate the coordinate value using two horizontal control points at each level. We then used this ratio to calculate two-dimensional coordinates (Ikeda et al., 2017, 2018, 2021). The coordinate values were digitally filtered using a Butterworth-type fourth-order low-pass filter. The cutoff frequency for the two-dimensional coordinates was 6 Hz (Butler et al., 2010; Hoogenboom et al., 2023; Ikeda et al., 2017). The linear and angular kinematics of the joints and segments were calculated from the smoothed coordinate data and the CM, and the inertial properties of each segment were calculated using body segment parameters for athletes (Ae, 1996). The shoulder, hip, knee joint, upper trunk, lower trunk, and upper leg angles were also calculated. The upper and lower trunk angles, divided by the inferior edge of the ribcage, were defined as the angles between the horizontal line and the upper and lower trunk, respectively.

The ground reaction forces in the vertical (Fz) and horizontal (Fy) directions for the left leg during the OHS were collected using a force plate at a sampling rate of 1000 Hz (9287C, Kistler Inc., Winterthur, Switzerland; total dimensions: 900 mm × 600 mm). The force and video were synchronized by recording the light of a synchronizer (PTS-110, DKH Inc., Itabashi-ku, Tokyo, Japan) at the signal.

The torque at the ankle, knee, and hip joints was calculated using the inverse dynamic method (Winter, 2009). The equations of motion for the foot, shank, and thigh were solved from the distal to the proximal end of the supporting leg using ground reaction force data. The OHS motion was divided into negative and positive phases based on the vertical velocities of the barbell and the center of mass (CM). The negative phase ranged from −0.1 m/s for the barbell to the lowest point of the CM, whereas the positive phase extended from the lowest point of the CM to 0 m/s for the barbell. The joint torque divided by BM was expressed as a relative value.

In the flexibility test, both the shoulder joint flexibility test (Figure 7) (Narita and Kaneoka, 2014) and the sit and reach test were performed. In the shoulder joint flexibility test, participants gripped a plastic bar with their elbows extended and rotated their shoulder joints from the top of the head to the back without bending their elbows. The distance between their hands was measured if participants could perform this movement without interruption. A shorter distance indicated higher shoulder rotational flexibility. The test was continued until the participant could not perform shoulder rotation. Narita and Kaneoka (2014) reported that the distance between the participants’ hands in the shoulder joint flexibility test was negatively correlated with the range of motion for shoulder extension, flexion, and external rotation, and that the value of the intraclass correlation coefficient (ICC) was more than 0.90.

**Figure 1:**
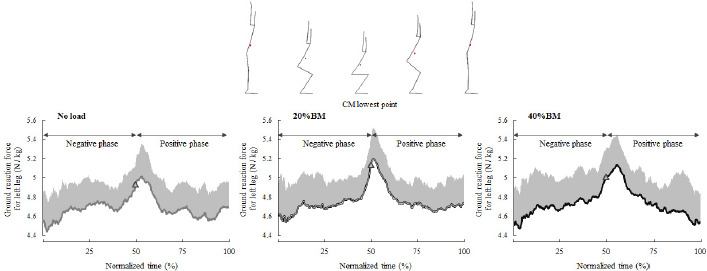
Changes in the average vertical ground reaction force of the left leg in the negative and positive phases of overhead squats. CM: center of mass; BM: body mass

**Figure 2:**
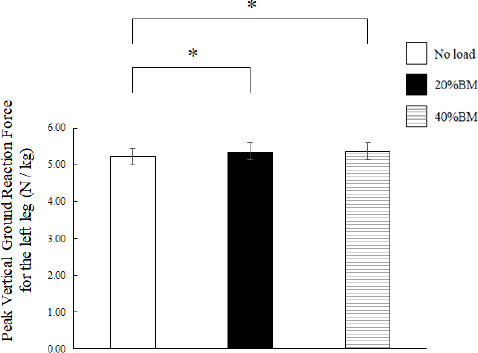
Comparison of peak vertical ground reaction force during OHS with no load, 20% BM, and 40% BM. OHS: overhead squat; BM: body mass; * Significant at p < 0.05

**Figure 3:**
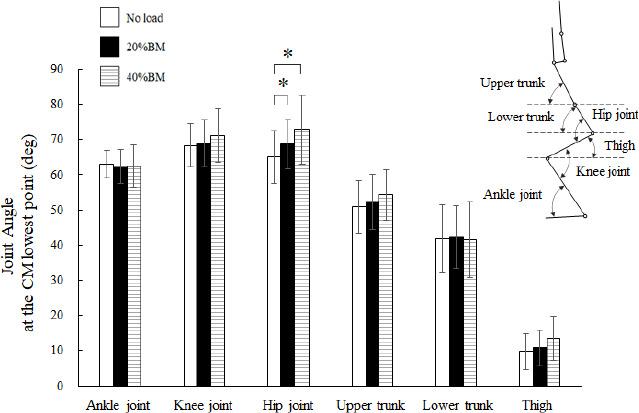
Comparison of joint angles at the CM lowest point during OHS with no load, 20% BM, and 40% BM. CM: center of mass; OHS: overhead squat; BM: body mass; * Significant at p < 0.05

**Figure 4:**
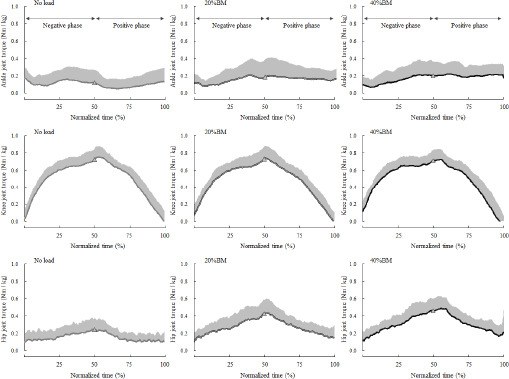
Changes in the average joint torque of the ankle, knee, and hip joints of the left leg in the negative and positive phases of overhead squats. CM: center of mass

**Figure 5:**
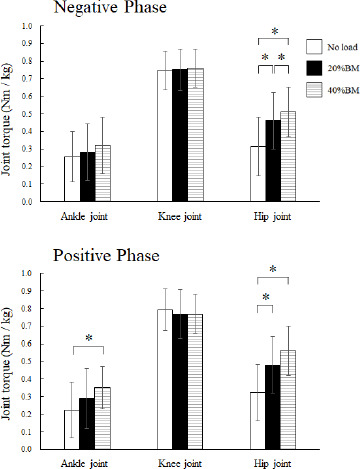
Comparison of the peak joint angles of the ankle, knee, and hip joints during the negative and positive phases of the OHS with no load, 20% BM, and 40% BM. BM: body mass; OHS: overhead squat; * Significant at p < 0.05

**Figure 6:**
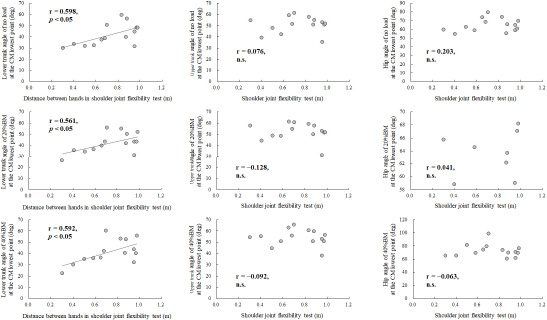
Relationship between the shoulder joint flexibility test score and lower and upper trunk angles at the lowest point of the CM during the OHS with no load, 20% BM, and 40% BM. BM: body mass; CM: center of mass; OHS: overhead squat

**Figure 7:**
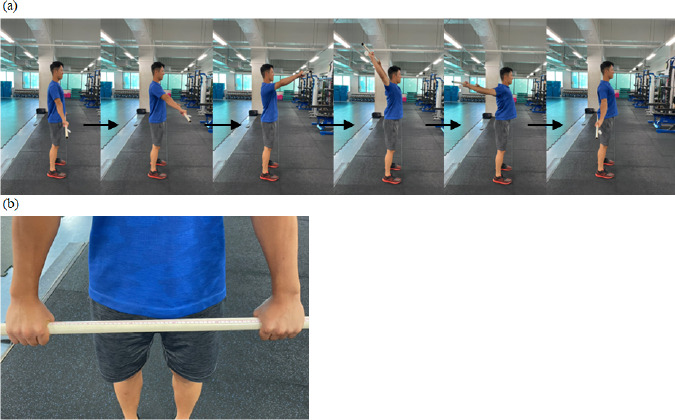
Shoulder joint flexibility test (a) and measurement of the distance between hands (b).

### Design and Procedures

Fourteen male college students who exercised regularly participated in this experiment to quantify the kinematics and kinetics of lower joint movements during the OHS with varying relative loads. Two-dimensional motion data and ground reaction forces were recorded for five consecutive OHS repetitions. The average data from the second and third trials out of five consecutive OHS repetitions were used for the analysis. Shoulder flexibility and sit and reach tests were conducted to investigate the correlation between shoulder joint and lower extremity flexibility and the kinetic and kinematic variables during the OHS. Each participant was instructed to complete two practice sessions and one experimental session to become acquainted with the OHS. In the two practice sessions, participants performed two or three sets of five consecutive OHS repetitions at three different loads (no load using a plastic pipe, 20% of body mass [BM], and 40% of BM) while maintaining a consistent motion speed controlled by a tempometer and the same depth and stance. During the experimental session, participants performed the OHS with three randomly assigned relative loads and flexibility tests. Two days were allowed between the last practice session and the experimental session to account for participants’ fatigue.

Participants performed a typical warm-up consisting of aerobic exercise using a stationary bike, which was executed at a set load and pedaling rate, and dynamic stretching, both for 10 minutes. After a 15-min rest interval, they practiced the OHS, took another 10-min rest interval, and then performed five consecutive OHSs on a force plate. The relative loads for the OHS were determined based on the BM of participants. The experiment involved three load conditions: no load, 20% of BM, and 40% of BM. Regarding the load for the OHS, the percentage of the one-repetition maximum load (%1RM) was initially planned to be used in the experiment. However, some participants expressed concerns about injuries to their shoulders and hips when using the heavy load in the practice session; therefore, the load was adjusted to a percentage of the BM instead. When determining the OHS load, some participants had difficulty completing five OHS repetitions at the same speed with a load equivalent to 60% of their BM during the practice sessions. Therefore, the OHS trial with 60% BM was excluded from the experiment. The trials of the OHS using three different loads were performed randomly, and participants were permitted a minimum of 3-min rest intervals between trials. All OHS repetitions were executed at a depth where the top of the thigh was parallel to the floor (Aspe and Swinton, 2014), as visually monitored by the researcher.

### Statistical Analysis

Values for each variable are presented as the mean ± standard deviation. Before the comparative analysis, the normality of the distribution of all variables was assessed using the Shapiro-Wilk test. Variables that were normally distributed among the three trials (no-load, 20% BM, and 40% BM conditions) were examined for differences using a one-way analysis of variance and Bonferroni’s post-hoc tests (parametric test). Non-normally distributed data were analyzed using a non-parametric (Friedman) test and the Bonferroni inequality test correction. Effect size was calculated using partial-eta squared for the parametric test (η^2^) and the results were interpreted as follows: small effect: 0.01–0.059, medium effect: 0.06–0.139, and large effect ≥0.14 (Cohen, 1998). Regarding the effect size of the non-parametric test, r_ef_ (r_ef_ = Z (test statistic) /√n) was calculated by the correction of the Bonferroni's inequality, and the effect size was interpreted as follows: small effect: 0.10–0.29, medium effect: 0.30–0.49, large effect ≥0.5 (Cohen, 1998). Pearson’s product-moment correlation coefficients (r) were used to investigate the relationships between the performance and kinetic and kinematic variables. All statistical procedures were conducted using SPSS Statistics 27 (IBM Corp., Armonk, NY, USA), and significance was set at *p* < 0.05.

## Results

The OHS trials took approximately 3.5–4 s to complete. Figure 1 shows the average peak vertical ground reaction force-time curves of the 14 participants and their respective SDs from start to finish for all OHSs. The temporal axis was standardized by the time from start to finish for all the OHSs. Nonparametric tests revealed significant differences in the relative peak vertical ground reaction force during the OHS (*p* < 0.01; Figure 2). Nonparametric multiple comparisons showed that the relative peak vertical ground reaction forces of the 20% (*p* = 0.004, r_ef_ = 0.77) and 40% BM (*p* = 0.011, r_ef_ = 0.68) conditions were significantly greater than those of the no-load condition. Regarding the kinematic variables, no significant differences were observed in the height at the lowest point of the CM among the no load (0.62 ± 0.03 m), 20% BM (0.62 ± 0.03 m), and 40% BM (0.63 ± 0.04 m) conditions. One-way analysis of variance revealed significant differences in the hip joint angle at the lowest point of the CM during the OHS (F = 16.189, *p* < 0.001, η^2^ = 1.25; Figure 3). Bonferroni’s post-hoc tests revealed that the hip joint angles of the 20% and 40% BM conditions were significantly greater than those of the no-load condition.

Figure 4 shows the mean joint torque-time curves in the 14 participants and their respective SDs from start to finish for all the OHSs. Non-parametric tests revealed significant differences in the relative joint torque for the hip (*p* < 0.001) in the negative phase and significant differences in joint torque for the ankle (*p* < 0.05) and the hip (*p* < 0.001) in the positive phase. Non-parametric multiple comparisons revealed that the relative hip joint torque of the 20% (*p* = 0.001, r_ef_ = 0.87) and 40% BM (*p* = 0.001, r_ef_ = 0.85) conditions was significantly greater than that of the no-load condition in the negative phase. Moreover, the relative hip joint torque of the 40% (*p* = 0.015, r_ef_ = 0.65) condition was significantly greater than that of the 20% condition in the negative phase and the relative hip joint torque of the 20% (*p* = 0.001, r_ef_ = 0.88) and 40% BM (*p* = 0.001, r_ef_ = 0.87) conditions was significantly greater than that of the no-load condition in the positive phase. The relative ankle joint torque of the 40% BM (*p* = 0.015, r_ef_ = 0.54) condition was significantly greater than that of the no-load condition in the positive phase (Figure 5).

Table 1 presents the correlation coefficients between the joint angles at the lowest point of the CM and the relative peak torque during the positive phase. The relative peak torque of the hip at all loads was negatively correlated with the angles of the upper and lower trunks at the lowest point of the CM. The relative peak torque of the knee with no load and 20%BM also negatively correlated with the knee angle at the lowest point of the CM.

**Table 1:** Correlation coefficients between joint angles at the CM lowest point and relative joint torque.

	Relative Joint Torque (Nm/kg)											
	No load				20%BM				40%BM			
Angles at the CM lowest point	Ankle	Knee	Hip		Ankle	Knee	Hip		Ankle	Knee	Hip
Ankle joint angle (deg)	0.094	−0.620*	0.410		−0.103	−0.458	0.545*		0.136	−0.653*	0.525
Knee joint angle (deg)	0.039	−0.623*	0.412		0.065	−0.718**	0.599*		−0.135	−0.350	0.399
Hip joint angle (deg)	−0.259	0.310	−0.503		0.007	0.181	−0.565*		0.532	0.430	−0.602*
Upper trunk joint angle (deg)	−0.436	0.519	−0.747**		−0.402	0.591*	−0.806**		0.199	0.436	−0.547*
Lower trunk joint angle (deg)	−0.359	0.166	−0.545*		−0.169	0.336	−0.717**		0.527	0.211	−0.652*

* , ** Correlations at p < 0.05 and p < 0.01, respectively

Figure 6 shows the correlation coefficients of the distance between the hands in the shoulder flexibility test and joint angles at the lowest point of the CM. The angle of the lower trunk at the lowest point of the CM was correlated with the distance between the hands in the shoulder rotation flexibility test, while no correlation was observed between the angles of the upper trunk (no load: r = 0.076, 20% BM: r = −0.128, 40% BM: r = −0.092.) and the hip (no load: r = 0.203, 20% BM: r = 0.041, 40% BM: r = −0.063) at the lowest point. Regarding the sit and reach test, the score of the sit and reach test was negatively correlated with the distance between the hands in the shoulder rotation flexibility test (r = −0.639, *p* < 0.05). Nevertheless, the score of the sit and reach test did not correlate with the angle of the lower trunk at the lowest point of the CM (no load: r = −0.396, 20%BM: r = −0.380, 40%BM: r = −0.345).

## Discussion

The present study was conducted to elucidate the kinematic and kinetic changes in the OHS resulting from increased loads and identify the key variables that affected the movement of the OHS. The relative peak torque of the hip for 20% and 40% BM in both the negative and positive phases was significantly greater than that under the no-load condition. In contrast, the relative peak torque of the knee did not exhibit a significant increase (Figure 5). The peak vertical ground reaction force at the lowest point of the CM also exhibited a change similar to that of the relative peak torque of the hip (Figure 2). Although few studies have focused on movement and joint torque during the OHS, Butler et al. (2010) suggested that participants who attained high FMS^TM^ scores could execute the OHS more deeply and exhibited greater knee and hip extension moments than those with low FMS^TM^ scores. Regarding the joint torque and muscle activity in the back squat movement with an increasing relative load, Manabe et al. (2003) also suggested that mean torque and work of the hip joint were significantly increased as the load increased from 60% and 75% to 90% of 1RM. Considering these findings, it is suggested that squat movements that increase the relative load may increase stress on the hip joint, regardless of the position of the load during the squat.

The relative peak torque of the hip joint was negatively correlated with the angles of the hip, upper trunk, and lower trunk at the lowest point of the CM (Table 1). These results suggest that forward-leaning of the upper and lower trunk and the small hip joint angle at the lowest point of the CM increased the relative peak torque of the hip joint during the positive phase. Regarding the trunk leaning during the back squat, Yavuz et al. (2015) found that the back squat, which displayed greater trunk leaning, showed higher electromyographic (EMG) activity in the semitendinosus than the front squat. Conversely, the front squat, which is characterized by an erect trunk, exhibited greater EMG activity in the vastus medialis. In addition, McLaughlin et al. (1977) pointed out that world-class powerlifters showed a more erect trunk and less horizontal barbell displacement than less-skilled participants. Schoenfeld (2010) also suggested that maintaining an upright posture was crucial to prevent lumbar injuries, because lumbar forces increased with greater forward leaning. Based on these findings and the results of this study, it is considered important in overhead squat training to maintain the angle of the upper and lower trunks as large as possible to reduce the load on the hip joints.

The angle of the lower trunk at the lowest point of the CM during the OHS was correlated with the distance between the hands in the shoulder flexibility test (Figure 6), whereas there were no significant correlations between the distance between the hands in the shoulder flexibility test and the angles of the upper trunk and the hip. In the flexibility tests, a significant correlation was observed between the distance of the hands in the shoulder flexibility test and the score of the sit and reach test; however, the score of the sit and reach test did not correlate with the angle of the lower trunk at the lowest point of the CM. These results indicate that a flexible shoulder joint may result in the lower trunk leaning forward during the OHS, and that control of the lower trunk angle affects the load on the hip joint extensor muscles. Therefore, athletes with flexible shoulder joints should pay attention to their lower trunk angle to prevent excessive hip extension torque.

During the movements in the OHS, the relative peak torque of the knee was greater than that of the hip and ankle joints, which suggested that the quadriceps femoris acted as an agonist muscle, which is consistent with previous squat studies (Figure 5) (Andrews et al., 1983; Isear et al., 1997; Manabe et al., 2004; Signorile, et al., 1994). The order of the relative peak torque of the lower extremities during the OHS was consistent with that in previous research (Hoogenboom et al., 2023). Escamilla (2001) reviewed previous studies and suggested that the squat was an effective exercise to employ during cruciate ligament or patellofemoral rehabilitation and exercise for athletes because it did not compromise knee stability and could enhance stability if performed correctly. Moreover, McCaw and Melrose (1999) emphasized the importance of compressive forces on the knee joint during the squat for the rehabilitation of ligament injuries. Based on the previous knowledge, for athletes aiming to strengthen the muscles around the knee joint and enhance their overall coordination abilities without increasing muscle hypertrophy, the OHS using a relatively low load will be an effective training method.

In squat training, the knee flexion angle often becomes a topic of discussion, depending on the individual. Klein (1961) examined the influence of the full-depth squat exercises and suggested that there might be a risk of collateral and cruciate ligament instability owing to the deep squat. Escamilla (2001) also recommended performing the parallel squat over the deep squat because the injury potential to the menisci as well as cruciate and collateral ligaments may increase with the deep squat. In the present study, using a parallel squat, a greater knee flexion angle at the lowest point of the CM produced greater knee extension torque during the positive phase (Table 1). Although the potential deleterious effects of deep squats remain controversial (Escamilla, 2001; Klein, 1961; Meyers, 1971), strength coaches and trainers might consider the possibility that excessive stretching of the lateral and medial collateral ligaments during deep squatting could potentially contribute to knee instability. In addition, since the overhead squat is a technically advanced exercise, it is possible that excessively fast movements cannot be properly supported by the knee and the trunk, which in turn can place significant stress on the joints and the neuromuscular system.

This study has several limitations. Regarding the load for the OHS, the percentage of the BM load was used in consideration of the injury risk of participants. However, %1RM should be used for a more precise measurement of the load. The experiment using the %1RM load should be considered for participants with sufficient upper and lower body strength and flexibility, such as weightlifters. Motion analysis was conducted in the sagittal plane, assuming that participants performed a symmetrical squat movement. The movements of the thigh and the lower leg in the transverse and frontal planes may have influenced the lifting motion (Hoogenboom et al., 2023). Future studies should consider examining the movement of the OHS using three-dimensional motion analysis. Moreover, EMG activities in the trunk and lower extremities were not investigated in this study; further research on muscle activities by EMG and joint torque during the OHS movement will provide crucial insights into training methods.

## Conclusions

The results of this study suggest that relative hip torque increases with an increasing load of the OHS and that the angles of the hip and the trunk at the lowest position of the CM are associated with the magnitude of the joint torque. Moreover, shoulder joint flexibility affected the lower trunk angle in the lowest CM position. These results indicate that a rapid increase in the load during OHSs may result in the overload of the hip joint, and that posture and movement in OHSs are related to shoulder joint flexibility. Based on these biomechanical findings, it may be possible for strength coaches and trainers to create effective training programs for athletes.
